# Essays on Life, Sleep and Pain, Etc.

**Published:** 1852-03

**Authors:** 


*Essays on Life, Sleep and Pain, etc.* By SAMUEL HENRY DICKSON, M. D., Professor of Institutes and Practice of Medicine in the Medical College of the State of South Carolina.

This is a very interesting book on a very interesting train of topics, involving the domains of physiology, hygiene, metaphysics, and ethics, and centering in that subject of all-absorbing fascination—"what 'tis we are, and we must shortly be." Our object in this notice is to express the very great gratification which the perusal of Dr. Dickson's Essays has afforded us. Designed, as the author informs us, rather for general than technical application, they are scarcely within the province of extended critical notice in a medical journal; and we have only to express our brief approval of what we feel satisfied must have already become one of the popular works of the day.

